# Stemless versus stemmed anatomic total shoulder arthroplasty: minimum 2- year follow-up of the global ICON and global unite systems

**DOI:** 10.1007/s00590-026-04796-w

**Published:** 2026-06-26

**Authors:** Roman Frederik Karkosch, Mara Knottnerus-Meyer, Tomas Smith, Hauke Horstmann, Spiros Tsamassiotis, Marc-Frederic Pastor

**Affiliations:** 1 Department of Orthopaedic Surgery, Hannover Medical School, DIAKOVERE Annastift, Hannover, Germany; 2 Department of Orthopaedic surgery, Klinikum Braunschweig, Braunschweig, Germany

**Keywords:** Glenohumeral osteoarthritis, Anatomic TSA, Stemless

## Introduction

Anatomic total shoulder arthroplasty (TSA) was first introduced by Neer et al. in 1955, primarily for treatment of proximal humerus fractures [1]. Neer´s initial prothesis design was a stemmed monobloc implant. Over time, TSA has continuously evolved and been refined to better replicate the natural anatomy of the glenohumeral joint [2, 3]. With an increase in implantations, humeral complications associated with stemmed protheses became evident. These included fractures, loosening, stress shielding, and periprosthetic fractures [4, 5].

Therefore, the necessity of the humeral stem was questioned, leading to the development of “stemless” resurfacing shoulder arthroplasty by Copeland and Levy [6]. Many companies have since developed stemless solutions, with the first being the TESS Biomet (Warsaw, IN, USA) in 2004. Stemless arthroplasty offers several advantages over traditional stemmed designs, including reduced surgical trauma due to less invasive techniques, potentially shorter operative times, and improved postoperative recovery [7]. Most important is the preservation of bone stock, which is especially beneficial for younger patients [8]. Studies have shown that stemless TSA can achieve comparable short-term survival rates compared to stemmed TSA, ranging from 83 to 98% [9, 10].

Despite these advantages, stemless shoulder arthroplasty remains challenging. Concerns have been raised about component malalignment as these designs require absolute surgical precision in order to avoid implant side complications such as overstuffing [11]. In the absence of a humeral stem metaphyseal bone stock is crucial for the implant’s stability and osteointegration of the humeral component. Nevertheless, the risk of certain stem-associated complications can be reduced, stemless designs are not immune to issues such as humeral fractures and the need for reinterventions [11].

Subsequently, it was the aim of this study to compare a modern stemless TSA (Global ICON™) and an established stemmed Solution (Global Unite™) in a retrospective design with at least 2 -year follow-up to generate outcome data and determine efficacy and safety of stemless TSA. It was hypothesized that regarding the Constant-Murley Shoulder Score stemless shoulder arthroplasty would not be inferior to the outcomes of stemmed TSA (H_1_). Moreover, revision rates and implant-specific complications were assumed to occur at equal rates (H_2_).

## Methods

### Study design

**Fig. 1:**
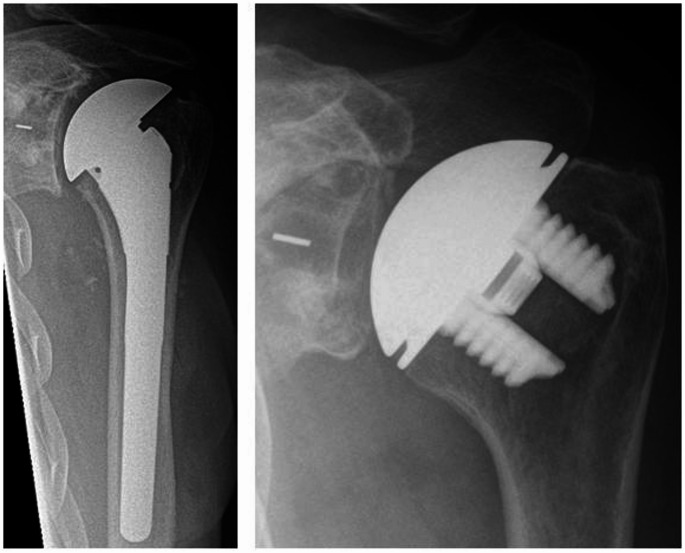
Display of Global Unite™ and Global ICON™ with Anchor Peg Glenoid

The present study was approved by the local ethics committee and designed in a retrospective design conducted by a single specialized orthopedic hospital. Included patients had undergone anatomic total shoulder arthroplasty (TSA) using either the Global ICON™ (Johnson & Johnson, New Brunswick, NJ, USA) stemless system or the Global Unite™ anatomic (Johnson & Johnson, New Brunswick, NJ, USA) stemmed system. All patients who received a stemmed humeral component were included in the study between June 2015 and November 2017. For the stemless humeral component, patients were followed up between June 2017 and March 2018, and between April 2019 and November 2019. The allocation of patients to stemmed versus stemless implants was not randomized but was primarily determined by the institutional transition from stemmed to stemless humeral components in 2017. Consequently, implant selection reflects a temporal change in surgical practice rather than purely patient-specific decision-making (see Fig. 1). All patients received an Anchor Peg Glenoid (Johnson & Johnson, New Brunswick, NJ, USA).

Each participant was contacted in advance by phone and provided written informed consent for a clinical and radiological follow-up examination. They were examined by a senior orthopedic specialist who was not the surgeon.

All participants underwent a clinical and radiological examination and were evaluated using established shoulder specific outcome measures. Standard plain X-rays were analyzed regarding radiolucent lines and implant positioning.

### Patient selection

Patients were selected based on predefined inclusion and exclusion criteria to ensure homogeneity in the study groups.

Inclusion criteria:

- Patients diagnosed with primary glenohumeral osteoarthritis requiring TSA.
- Age ≥ 18 years at the time of surgery.
- No previous shoulder replacement or significant trauma history.
- Adequate bone stock to support prosthetic implantation.

Exclusion criteria:

- Post-traumatic arthritis or prior proximal humerus fractures
- Neurological disorders affecting shoulder function
- Presence of inflammatory joint diseases such as rheumatoid arthritis
- Any previous rotator cuff surgery or prior shoulder hemiarthroplasty

### Surgical technique

All surgeries were performed under general anesthesia. Patients were placed in the beach-chair position, and a standardized deltopectoral approach was used for both implants.

For stemmed prostheses, humeral head osteotomy was performed, followed by intramedullary canal preparation using sequential broaching. The Global Unite™ prosthesis was then press-fitted.

For stemless prostheses, the humeral head was resected according to patient-specific anatomical landmarks in the corresponding retroversion and inclination using the saw guide. After the resection of the humeral head the thumb-test was performed to check the bone quality. A central metaphyseal cavity was prepared for the Global ICON™ implant, which was then secured with multiple fixation points to achieve primary stability without the use of cement.

Glenoid preparation and implantation were identical in both groups, with an Anchor-Peg polyethylene glenoid component being used. The subscapularis tendon was repaired using a standard tendon-to-bone technique with DYNACORD™ sutures (Johnson & Johnson, New Brunswick, NJ, USA) passed through transosseous drill holes placed in the anatomical footprint prior to implantation of the humeral head component. No major changes in the repair technique occurred during the study period.

All patients followed a standardized postoperative rehabilitation protocol that did not differ between the intervention groups.

### Data collection

Clinical data was gathered through both objective and subjective assessments. The following validated scoring systems were used:

- Constant Murley Shoulder Score (Primary outcome measure)
- Simple Shoulder Test (SST)
- American Shoulder and Elbow Surgeons Score (ASES)
- Oxford Shoulder Score (OSS)
- SF-36 Health Survey

Radiographic evaluations included:

- Presence of radiolucent lines
- Signs of stress shielding
- Humeral head subluxation assessment

In addition to clinical outcome measures, baseline patient characteristics were collected to assess comparability between groups. These included age, sex, body mass index (BMI), body weight, and height. Furthermore, where available, anatomical parameters such as glenoid morphology were assessed using preoperative imaging.

### Statistics

All statistical analyses were conducted using SPSS Statistics (Version 29.0, IBM, Armonk, NY, USA). Descriptive statistics were reported as means and standard deviations. To test the main hypothesis, the results of the Constant Murley Score for the groups under study were compared using an independent-samples t-test, and the noninferiority of the stemless implant was assessed. A non-inferiority margin of 10 points between the two groups was assumed based on data from the literature [12]. The confidence level of this study was set at 95%. To examine the influence of additional factors (age, body weight, height, psychological summary score of the SF-36 2.0, OSS) on the Constant Murley Score outcome, a multiple linear regression analysis was conducted.

A t-test for independent samples was used to compare clinical scores between the two groups. The chi-square or Fisher’s exact test was applied for categorical variables. A *p*-value < 0.05 was considered statistically significant.

## Results

### Clinical outcomes

A total of 48 patients were included in the final analysis, comprising 22 patients treated with a stemless humeral component and 26 patients treated with a stemmed humeral component. Baseline demographic characteristics were largely comparable between groups. The follow-up duration ranged from 27 to 63 months in the stemless group and from 54 to 81 months in the stemmed group.

Mean age at follow-up was 69.5 ± 8.8 years in the stemless group and 73.2 ± 8.9 years in the stemmed group, while mean age at surgery was 65.8 ± 8.7 and 67.7 ± 9.0 years, respectively. No statistically significant differences were observed for age-related parameters. The mean follow-up duration, however, was significantly longer in the stemmed group (67.2 ± 8.2 months) compared with the stemless group (44.5 ± 11.8 months; *p* < 0.001) (compare Table 1).

**Table 1:** Patient characteristics and clinical outcomes

Domain	Variable	Stemless TSA (n = 22)	Stemmed TSA (n = 26)
Diagnosis	Primary glenohumeral osteoarthritis	19	23
	Instability-related osteoarthritis	3	3
Demographics	Age at follow-up, years (mean ± SD)	69.50 ± 8.83 (22)	73.19 ± 8.90 (26)
	Age at surgery, years (mean ± SD)	65.77 ± 8.69 (22)	67.65 ± 8.97 (26)
	Length of follow-up, months (mean ± SD)	44.50 ± 11.81 (22)	67.23 ± 8.23 (26)
Sex	Male	9	17
	Female	13	9
Anthropometrics	Weight, kg (mean ± SD)	72.91 ± 10.73	83.92 ± 18.25
	Height, cm (mean ± SD)	167.09 ± 8.29	172.88 ± 9.43
	Body mass index, kg/m^2^ (mean ± SD)	26.07 ± 3.14	27.92 ± 4.90
Side of surgery	Left	7	11
	Right	15	15
Constant score	Operated side (mean ± SD)	76.95 ± 17.05	80.12 ± 8.16
	Age-adjusted (mean ± SD)	89.05 ± 18.34	91.04 ± 9.66
	Contralateral side (mean ± SD)	83.14 ± 7.88	85.62 ± 7.08
SST	Simple Shoulder Test score (mean ± SD)	81.06 ± 24.83	74.68 ± 19.07
ASES	Operated side (mean ± SD)	86.50 ± 12.24	89.92 ± 17.02
OSS	Oxford Shoulder Score (mean ± SD)	54.09 ± 8.99	54.46 ± 5.31
SF-36	Physical Component Summary (mean ± SD)	50.90 ± 15.12	48.43 ± 17.32
	Mental Component Summary (mean ± SD)	74.66 ± 12.38	79.21 ± 13.50
Patient-reported outcomes	Patient satisfaction (yes)	20	26
Pain	Rest pain (yes)	2	7
	Activity-related pain (yes)	6	10
Medication	Use of analgesics (yes)	4	3
Rehabilitation	Able to work (yes)	9	5
	Retired	12	21
	Overhead activities possible (yes)	21	24
	Return to preoperative sports level (yes)	20	22
Surgical parameters	Surgical time, minutes (mean ± SD)	74.18 ± 14.60	81.69 ± 15.49
Complications	Occurrence (yes)	3	0

Values are presented as mean ± standard deviation or absolute numbers.

TSA, total shoulder arthroplasty; SST, simple shoulder test; ASES, American shoulder and elbow surgeons score; OSS, oxford shoulder score; SF-36, short form-36 health survey.

Baseline demographic characteristics demonstrated relevant differences between groups. While age at surgery and follow-up were comparable, the stemless group included a higher proportion of female patients (59.1% vs. 34.6%), whereas the stemmed group was predominantly male (65.4%). Additionally, patients in the stemmed group had higher mean body weight (83.9 ± 18.2 kg vs. 72.9 ± 10.7 kg), height (172.9 ± 9.4 cm vs. 167.1 ± 8.3 cm), and BMI (27.9 ± 4.9 kg/m^2^ vs. 26.1 ± 3.1 kg/m^2^). These differences indicate that complete baseline equivalence between groups cannot be assumed. In both groups, the right shoulder was more frequently operated on.

Functional outcomes assessed by the Constant Score demonstrated no significant differences between groups. The mean Constant Score of the operated shoulder was 77.0 ± 17.1 in the stemless group and 80.1 ± 8.2 in the stemmed group (p = n.s.). Analysis of Constant Score sub scores (pain, activities of daily living, range of motion, and strength) revealed no statistically significant intergroup differences.

Regarding H_1_ the mean difference in the Constant-Murley Shoulder Score between the stemless and stemmed group was − 3.16 points. While the independent t-test showed no significant difference (p = n. s.), the lower bound of the 95% confidence interval (− 11.29 to 4.96) slightly exceeded the predefined non-inferiority margin of 10 points. Therefore, while no clinical inferiority was observed in the mean values, formal statistical non-inferiority could not be strictly established within this sample size.

Regarding H_2_, postoperative complications occurred in 13.6% (n = 3) of the stemless group, while no complications were observed in the stemmed group. Fisher’s exact test showed no statistically significant difference between the two implant models (p = n. s.). Thus, while the complication rates did not differ significantly, a numerical trend was observed.

**Fig. 2:**
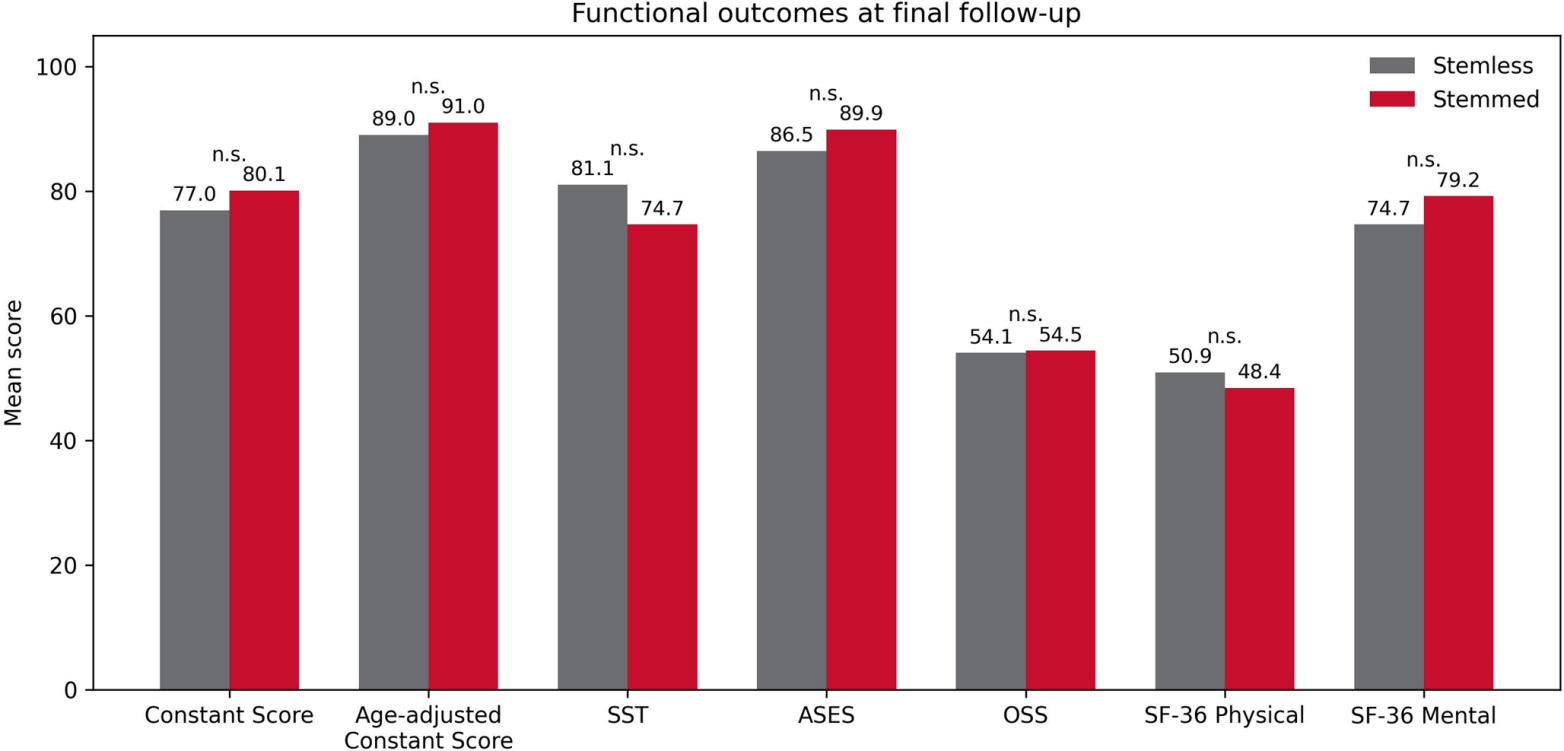
Comparison of functional outcome scores at final follow-up between stemless and stemmed humeral components. Data are presented as mean values

Patient-reported outcome measures showed comparable results between cohorts. The Simple Shoulder Score, ASES score, Oxford Shoulder Score, and SF-36 physical and mental component summary scores did not differ significantly between groups (Fig. 2 provides a comprehensive display of all outcome measures). Overall patient satisfaction was high, with 90.9% of patients in the stemless group and 100% of patients in the stemmed group reporting satisfaction with the surgical outcome. All patients claimed that they would choose to undergo the procedure again.

At follow-up, pain at rest and during activity was infrequently reported in both cohorts. A higher proportion of patients in the stemmed group reported residual rest pain (26.9% vs. 9.1%). Analgesic consumption was low and comparable between groups. Most patients were retired at follow-up, and the majority reported preserved ability to perform overhead activities and return to their previous level of sports participation.

### Radiographic findings

Radiographic evaluation showed no humeral radiolucency in the stemless group. In contrast, radiolucent lines around the humeral stem were observed in 69.2% of patients treated with a stemmed implant. They varied in size from 0.5 to 2.0 mm concentrating in zone 8 (below humeral head component) and zones 3–5 (around the tip of the stem) according the description of Merolla et al. [13].

Glenoid radiolucency was rare and similarly distributed between groups. Stress shielding was observed in 19.2% of stemmed implants, but was not observed in the stemless group. No significant differences were observed between the groups in terms of humeral head positioning or implant alignment. Patients individual anatomy and offset were reconstructed according to the best fit circle principle. Both groups demonstrated stable postoperative positioning, without cases of severe malalignment, dislocation or immediate implant failure. Upward migration of the humeral head did not occur.

The distribution of glenoid types was comparable between the two groups. In the stemmed (Unite) group, the majority of patients presented with type A glenoids (A1: n = 12; A2: n = 9), with fewer cases of B-type (B1: n = 3; B2: n = 1), type C (n = 1), and no type D glenoids. Similarly, in the stemless (ICON) group, type A glenoids were predominant (A1: n = 9; A2: n = 8), followed by B-type glenoids (B1: n = 1; B2: n = 1; B3: n = 3), with no type C or D glenoids observed.

Overall, no relevant differences in glenoid morphology distribution were identified between groups, suggesting comparable preoperative joint anatomy with respect to glenoid wear patterns. No statistically significant differences were observed between groups (p = n.s.). Calcar resorption was not specifically evaluated in this dataset and therefore cannot be reported (Table 2).

**Table 2:** Incidence of radiolucent lines around the humeral and glenoid components in stemless (Global ICON™) and stemmed (Global Unite™) total shoulder arthroplasty

Radiolucent lines	Global Icon™ (n = 22)	Global unite™ (n = 26)	Overall (n = 48)
Glenoid	1 (4.5%)	4 (15.4%)	5 (10.4%)
Humeral	0 (0%)	18 (69.2%)	18 (37.5%)
	1 (2.1%)	22 (45.8%)	

### Operative time and complications

Mean operative time was 74.2 ± 14.6 min in the Global ICON™ group and 81.7 ± 15.5 min in the stemmed group, with a statistically significant difference (*p* = 0.046). Complications with the need of revision occurred in three patients (13.6%) in the stemless group, whereas no complications were observed in the stemmed group; this difference did not reach statistical significance. Issues observed in the stemless cohort included a traumatic subscapularis tendon rupture (n = 1, arthroscopic repair), postoperative stiffness (n = 1, arthroscopic arthrolysis) and persisting pain due to an os acromiale (n = 1, screw fixation). Each case was treated surgically. No revision surgeries were required in the stemmed group. Periprosthetic joint infections did not occur throughout the follow-up.

## Discussion

The present study compared the clinical and radiographic outcomes of a modern stemless humeral implant (Global ICON™) with those of a conventional stemmed prosthesis (Global Unite™) in patients undergoing anatomic total shoulder arthroplasty (TSA) for primary glenohumeral osteoarthritis.

The most important finding of this investigation is that both stemless and stemmed TSA demonstrated satisfactory clinical and radiographic outcomes at follow-up. However, due to the absence of preoperative functional data and baseline differences between groups, a definitive comparison between implant types remains limited.

In line with the observed findings, both implant designs achieved similar functional outcomes at follow-up. These results are consistent with current literature, which likewise reports comparable clinical and radiographic outcomes between stemmed and stemless total shoulder arthroplasty designs [2, 14]. Previous comparative reviews of stemmed and stemless total shoulder arthroplasty (TSA) have reported mean Constant Scores of approximately 74.8 points for stemmed designs and 76.9 points for stemless designs [14]. These values are comparable to the findings of the present study, in which mean Constant Scores of 80.1 points for stemmed TSA and 77.0 points for stemless TSA were observed. Longitudinal analyses have demonstrated a gradual improvement in Constant Scores over time, with increases of approximately five points between 12-month and longer-term follow-up [10]. Similarly, a meta-analysis by Looney et al., including 229 patients treated with stemmed TSA and 358 patients treated with stemless TSA, reported comparable clinical outcomes between implant designs [15]. Further, our findings were aligned with the registry studies. Issa et al. reported similar short-term survival and patient-reported outcomes for the stemless and stemmed TSA in the Danish registry [16]. Nying et al. confirmed comparable low revision rates between two implant designs in a larger multi-national registry collaboration [17]. Collectively, these data support our findings that stemless arthroplasty provides a safe alternative to stemmed design.

Radiolucent lines around the humeral component were observed in 69.2% of stemmed implants, whereas no humeral radiolucency was detected in the stemless group. This marked difference should be interpreted with caution, as the stemmed cohort had a significantly longer follow-up period, which may increase the likelihood of detecting such radiographic changes.

In all cases, radiolucent lines were less than 2 mm in extent and were not associated with clinical symptoms.

Previous studies investigating stemless TSA from various manufacturers have reported rates of humeral radiolucent lines ranging from 7.1 to 18.4% [2, 18]. In contrast, Sanchez-Sotelo et al. described radiolucent lines in 55.6% of stemmed TSA cases at a mean follow-up of 4.1 years [19]. The clinical relevance of radiolucent lines has been suggested to be limited unless multiple zones or distal zones are involved [20]. In the present cohort, no revision surgery was required due to humeral component loosening. As established in other joint arthroplasties, the occurrence of radiolucent lines is multifactorial and influenced by patient-related factors, such as body mass index, age, and bone quality, as well as implant design and fixation technique [21]. Further, in the group with the stemmed humeral component more radiolucent lines were observed on the glenoid (15.4% vs. 4.5%). This could be explained by the longer follow-up period for this group. However, these radiolucent lines did not appear to affect the clinical outcomes.

Stemless TSA offers several theoretical and practical advantages, including reduced intraoperative blood loss and preservation of humeral bone stock due to metaphyseal fixation [22, 23]. This fixation concept may facilitate easier humeral component removal during revision procedures, particularly when revision to reverse shoulder arthroplasty is required, potentially improving revision surgery outcomes [24]. Nevertheless, adequate metaphyseal bone quality remains a critical prerequisite for stemless TSA. In previous studies, insufficient metaphyseal bone stock necessitated intraoperative conversion to stemmed TSA in up to 83% of cases [2, 25]. Moreover, this study demonstrated significantly shorter surgeries for the stemless implant by an average of 7.5 min (*p* = 0.046) suggesting a slight efficiency advantage for the stemless implant.

A major strength of the present study is the inclusion of rare short – to mid-term follow-up data for the Global ICON™ stemless shoulder arthroplasty system, with a mean follow-up of 44.5 months. Currently, only two other studies reported two-year follow-up outcomes for this implant [26, 27]. Their data demonstrated significant improvements in the WOSS, with no implant-related complications reported [26]. These results are consistent with the present findings, which showed a mean WOSS of 54.1 points.

The increased complication rate observed in the stemless group warrants further investigation. Yet, traumatic subscapularis rupture and persisting pain due to an os acromiale should be interpreted as free of device complications. While the literature reports a deep infection rate of up to 2.2% for stemless implants, this was not an issue in this investigation (0%) [14].

Additionally, the reduced complexity of revision surgery associated with stemless prostheses may represent a clinically relevant advantage that is not fully captured in short- to mid-term outcome analyses. Future studies should therefore include larger patient cohorts and extended follow-up periods to assess long-term implant survival, osteointegration, and late complications.

### Limitations

Nevertheless, several limitations of this study should be acknowledged. First, the retrospective design introduced potential selection bias, as the choice between a stemless and a stemmed prothesis was based on the surgeon’s choice and time rather than randomization.

However, differences in baseline characteristics, including sex distribution and anthropometric parameters, indicate that comparability between groups is limited. To minimize the selection bias, patients were included on the specific consecutive time periods. Notably, the substantially longer follow-up duration in the stemmed group may have contributed to the higher observed rates of residual pain and radiographic changes, including radiolucent lines.

Second, the overall sample size was relatively small, which may reduce the statistical power to detect subtle differences between the implant groups. Third, the follow-up duration differed between the two cohorts, with a longer mean follow-up in the stemmed group. This difference may influence the detection of radiographic changes and late complications. Furthermore, preoperative clinical outcome scores were not available, precluding an analysis of postoperative improvement relative to baseline function. Finally, the introduction of the Global ICON implant during the study period may have been associated with a learning curve that could have influenced early outcomes.

## Conclusion

Despite these limitations, the findings of the present study support the growing body of evidence suggesting that stemless anatomic total shoulder arthroplasty provides clinical outcomes comparable to those of traditional stemmed implants**,** while offering potential advantages with respect to bone preservation and revision surgery. Future prospective studies with larger patient cohorts and longer follow-up durations are required to further evaluate long-term implant survival, osseointegration, and complication profiles associated with stemless humeral fixation.

## Data Availability

The data sets generated and/or analyzed during the current study are available from the corresponding author on reasonable request.
